# Depressive and Anxiety Symptoms in Women with Polycystic Ovary Syndrome: A Meta-Analysis

**DOI:** 10.3390/jcm15103582

**Published:** 2026-05-07

**Authors:** Katarzyna Stańczyk, Olga Łopacińska, Dominika Kędzia, Oliwia Gawlik-Kotelnicka

**Affiliations:** 1 Department of Sports Medicine, Medical University of Lodz, Pomorska 251, 92-213 Łódź, Poland; 2 Faculty of Medicine, Medical University of Lodz, Al. Tadeusza Kościuszki 4, 90-419 Łódź, Poland; dominika.kedzia@student.umed.lodz.pl; 3 West Pomeranian Center of Treating Severe Burns and Plastic Surgery, Niechorska 27, 72-300 Gryfice, Poland; olga.lopacinska@student.umed.lodz.pl; 4 Department of Affective and Psychotic Disorders, Medical University of Lodz, Czechosłowacka 8/10, 92-216 Łódź, Poland; oliwia.gawlik@umed.lodz.pl

**Keywords:** depression, anxiety disorders, endocrine disease, patient

## Abstract

**Background/Objectives:** Polycystic ovary syndrome (PCOS) is the most commonly diagnosed endocrine disorder in women of reproductive age, with a prevalence estimated at 4–20%. Among the conditions often co-occurring with PCOS are depressive and anxiety disorders. The aim of this study was to determine the prevalence of depressive and anxiety symptoms in women with PCOS. **Methods:** A literature search was conducted using PubMed/MEDLINE, Google Scholar, and Directory of Open Access Journals databases for studies published before May 2025. The analysis was conducted on a group of 5857 women (study group: 3610; control group: 2247) obtained from 35 studies, which met the inclusion criteria. **Results:** The prevalence of depressive symptoms in PCOS patients and the controls, according to the BDI, HADS-D, Mini-NPI and PHQ assessment tool, was 42.11% (95% CI: 32.6–52.2) vs. 13.62% (95% CI: 8.4–21.5; *p* < 0.001); 28.90% (95% CI: 20.7–38.8) vs. 15.80% (95% CI: 11.3–21.7; *p* = 0.010); 59.10% (95% CI: 30.9–82.4) vs. 65.90% (95% CI: 24.2–92.1; *p* = 0.792); and 26.50% (95% CI: 8.0–59.9) vs. 9.10% (95% CI: 1.8–35.1; *p* = 0.255). The prevalence of anxiety symptoms in the PCOS group and the control group, according to the HADS-A assessment tool, was 48.25% (95% CI: 36.1–60.6) vs. 31.40% (95% CI: 18.8–47.4; *p* = 0.098), respectively. **Conclusions:** The study confirms the higher prevalence of depressive symptoms in women with PCOS compared to the general population. These findings indicate the necessity of conducting psychiatric examinations and providing support for women with PCOS.

## 1. Introduction

Polycystic ovary syndrome (PCOS) is the most prevalent endocrine disorder in women of reproductive age, affecting 4–20% of the female population worldwide [1]. The difference may be due to the diagnostic criteria used. The first standard diagnostic criteria were established in 1990 as a result of a meeting of experts in medical and reproductive endocrinology sponsored by the National Institutes of Health (NIH). Those criteria, which included clinical and/or biochemical hyperandrogenism and ovulatory dysfunction, with exclusion of secondary causes, became known as the NIH criteria [2]. The prevalence of PCOS remained stable at 5–8% according to the abovementioned criteria [3]. Then, in 2003, a clause concerning the image of polycystic ovaries in ultrasound examination was introduced into the criteria. It occurred as a result of a meeting sponsored by the European Society for Human Reproduction and Embryology and the American Society for Reproductive Medicine (ASRM), which was organized in Rotterdam. At that time, two of the three features had to be met to diagnose PCOS [4]. Under the Rotterdam criteria, the prevalence of the disorder was 12–18% [5]. Currently, it is recommended to use the modified Rotterdam criteria, revised in 2023. After the exclusion of secondary causes, two of the following three features are required: (1) ovulatory disorders (oligoovulation or anovulation); (2) clinical and/or biochemical hyperandrogenism; and (3) image of polycystic ovaries (≥20 follicles in at least one ovary, or volume of at least one ovary ≥ 10 mL if older technology is used, or limited visibility) on ultrasound, or elevated serum levels of Anti-Müllerian Hormone (AMH). The 2023 recommendations do not specify a single, global AMH threshold, but instead recommend using assay-specific and population-specific cut-offs. As reported, AMH levels are typically 2–3 times higher in women with PCOS compared to the control group [6].

However, despite the high prevalence of PCOS, the pathogenesis of this disorder remains unclear. The relationship between biochemical hyperandrogenism and insulin sensitivity in target tissues is given particular attention, as well as functional defects in the insulin receptor. The latter leads to impaired activation of the phosphoinositide kinase (PI3K) signalling pathway as a result of excessive serine phosphorylation and insufficient tyrosine phosphorylation. Impaired intracellular glucose transport promotes the development of insulin resistance, which, together with compensatory hyperinsulinemia and hyperandrogenism, is one of the direct causes of reproductive dysfunction in PCOS women [7]. Other possible clinical manifestations of hyperandrogenism, mainly elevated testosterone concentrations, may include acne, androgenic alopecia, and hirsutism [8]. The skin-related problems listed above, infertility, and obesity can lead to emotional distress, low self-esteem, and social stigmatization, resulting in profound psychological implications [9]. According to research, women with PCOS are at increased risk of developing such neuropsychiatric disorders as depression, anxiety disorders, bipolar disorder, and obsessive–compulsive disorder [10,11]. It is estimated that they are 3 to 8 times more likely to develop depression than the general female population [12], and a multitude of studies linking PCOS to depression and anxiety emphasize a significant comorbidity that can negatively impact quality of life [13]. As defined by the American Psychiatric Association, major depressive disorder is a depressive episode with ≥5 symptoms, including at least depressed mood or markedly diminished interest or pleasure, occurring most of the day, nearly every day, for ≥2 weeks, and causing clinically significant distress [14], while anxiety is a feeling of tension, disturbing thoughts, and physical changes [15]. Although the mechanisms underlying the association of PCOS with anxiety and depression disorders are still under debate, many studies indicate that stress has a crucial role in the etiology of these disorders [12,16]. This can be supported by the fact that PCOS patients experience higher levels of perceived stress than the general population [17] and were hospitalized twice as often due to stress and self-harming behaviour [18]. The potential impact of PCOS symptoms, the effect of hormonal alterations, or the combined effect of both factors on the onset of depression and anxiety were also indicated [17,19,20,21], while one study focused on the impact of neurotransmitters [10]. It has been observed that in PCOS, the concentrations of inhibitory neurotransmitters such as serotonin, dopamine, gamma-aminobutyric acid, and acetylcholine are reduced, while the concentration of glutamate, the main stimulant of gonadotropin-releasing hormone and LH, is elevated. These neurotransmitter alterations may play a significant role in mood disorders [10]. Finally, as recent reports indicate, one of the most common metabolic dysfunctions in PCOS women, insulin resistance, may directly contribute to the development of psychiatric disorders. It has been observed that patients with depression have greater fluctuations in insulin levels than the control group, and during acute episodes, the insulin sensitivity of target tissues decreases. Although the hypothesis that insulin resistance is a particular metabolic subtype of depression has not been conclusively confirmed, it has been proven that insulin concentration and the HOMA-IR (homeostatic model assessment for insulin resistance) index may be state biomarkers of depression. Monitoring these parameters, especially in atypical depression, provides an opportunity to stratify patients and guide the selection of personalized lifestyle interventions and pharmacological therapy, which can substantially increase the success rate of the treatment [22].

Prior to a comprehensive psychiatric examination, the occurrence and severity of symptoms of depression and anxiety can be screened using standardized questionnaires, such as the Beck Depression Inventory (BDI), the Hospital Anxiety and Depression Scale-Depression (HADS-D), the Mini-International Neuropsychiatric Interview (Mini-NPI), the Patient Health Questionnaire (PHQ), and the Hospital Anxiety and Depression Scale-Anxiety (HADS-A). The results obtained in the survey do not constitute a basis for diagnosis, but may indicate specific areas requiring further investigation.

This study conducted a meta-analysis of the risk association between the occurrence of depression or anxiety symptoms and PCOS, compared to the general adult female population, to provide medical evidence for research on the treatment of this disorder.

## 2. Materials and Methods

### 2.1. Search Methodology

The study was designed in accordance with the Preferred Reporting Items for Systematic Reviews and Meta-Analyses (PRISMA) [23,24]. The PRISMA 2020 checklist is provided as Supplementary Material (Table S1). The study protocol was not registered in the International Prospective Register of Systematic Reviews (PROSPERO). A systematic literature search was conducted until May 2025 using three databases: PubMed/MEDLINE, Google Scholar, and Directory of Open Access Journals (DOAJ). A literature search was performed using the following keywords: “polycystic ovary syndrome”, “polycystic ovarian syndrome”, “PCOS”, “depression”, “depressive disorders”, “anxiety”, “anxiety disorders”. Moreover, keywords were entered into the keyword search tool as follows: (“polycystic ovary syndrome” OR “polycystic ovarian syndrome” OR “PCOS”) and (“depression” OR “depressive disorders” OR “anxiety” OR “anxiety disorders”).

### 2.2. Eligibility Criteria

Both the inclusion and exclusion criteria were used for the systematic review. The following inclusion criteria were applied:

1. The study included adult women over 18 years.
2. The study included pre-menopausal women.
3. The study included women diagnosed with polycystic ovary syndrome on the basis of criteria developed by the National Institutes of Health (NIH), the Rotterdam Consensus or others.
4. The study group included 30 or more patients.

The exclusion criteria were framed as follows:

1. Case report, narrative review, systematic review or meta-analysis studies.
2. Studies not written in English.
3. Studies conducted among adolescent or post-menopausal women.
4. Studies conducted among pregnant or breastfeeding women.
5. Studies assessing the effect of the drug on symptoms of depression or anxiety.

Following a rigorous review of the extant literature, it was determined that research conducted among adolescent or post-menopausal women would be excluded on the basis of their low availability in the existing research to ensure data consistency. The selection of specific screening tools incorporated into the analysis was predicated on the availability of the relevant data in the extant literature, as well as ensuring the efficacy of the statistical analysis outcomes, with the requisite sample size being a fundamental consideration. The search and selection of studies were designed according to the population, intervention, comparison, outcomes, and study design (PICOS) strategy, presented in Table 1.

### 2.3. Study Objectives

This meta-analysis aims to quantitatively synthesize the pooled prevalence of depressive and anxiety symptoms, assessed using standardized tools, among women diagnosed with polycystic ovary syndrome, compared to the general adult female population. In the studies we included in our analysis, depressive symptoms were assessed using the BDI, HADS-D, Mini-NPI, and PHQ screening tools, while anxiety symptoms were evaluated with the HADS-A screening tool. These standardized self-assessment questionnaires provide an opportunity to evaluate various depressive symptoms, and the consistency of findings across instruments increases confidence in the observed association between PCOS and depression. Additionally, the study seeks to compare these prevalence estimates with those observed in a control population of women without PCOS, thereby elucidating the psychological burden associated with PCOS and informing targeted clinical interventions.

### 2.4. Study Selection

**Figure 1:**
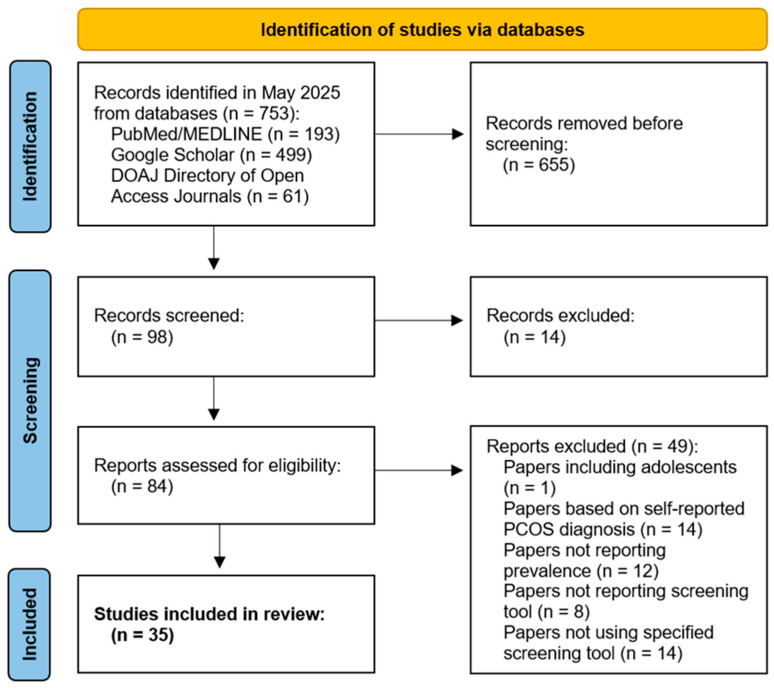
The PRISMA chart describing the selection process for the systematic review.

The systematic review of literature was completed on 2 May 2025. As 669 studies did not meet PRISMA criteria, duplicative or not available in full text, 84 eligible studies were finally selected. Subsequently, 53 studies were excluded due to the following reasons: included adolescent patients (*n* = 1); were based on self-reported PCOS diagnosis (*n* = 14); did not report the prevalence of depressive or anxiety symptoms (*n* = 12); did not report the screening tool used to investigate depressive or anxiety symptoms (*n* = 8); did not employ selected screening tool, such as the BDI, HADS-D, Mini-NPI or PHQ to assess symptoms of depression or the HADS-A to assess symptoms of anxiety (*n* = 14). This reduced the total number of eligible studies to 35, including 35 investigating symptoms of depression and 15 examining symptoms of anxiety. For each of the analyzed papers, the following information was extracted: the first author, year of publication, country where the research was conducted, and the size of the study sample. The review attempted to extract the following information from the papers included in the analysis: the criteria used for the diagnosis of PCOS, information on whether the diagnosis of PCOS was confirmed by a specialist or reported by the patient, variables used to match PCOS subjects and controls, mean age and BMI in the study and control groups, the tools used to investigate the presence of depressive and/or anxiety symptoms, the medical exclusion criteria for the study, the prevalence of depressive and/or anxiety symptoms, the mean score obtained for the severity of depressive and/or anxiety symptoms, the level of statistical significance of the obtained outcome (Tables S2 and S3 in the Supplementary Material). The process of selecting articles for review, following PRISMA principles, is presented in Figure 1.

### 2.5. Statistical Analysis

To estimate the prevalence of depression and anxiety among women with PCOS compared to controls, a meta-analysis of proportions was conducted [25]. The proportion of depression and anxiety cases, defined as the number of events divided by the total sample size per study group, was analyzed. The logit transformation was applied to stabilize the variance of proportions and ensure that pooled estimates remained within the interval [0, 1] after back-transformation [26].

The primary analysis employed a Generalized Linear Mixed Model (GLMM) approach to model the binomial outcome of depression and anxiety directly, using a logistic link function [27]. This method was selected for its robustness in handling small sample sizes (e.g., as low as 18) and extreme proportions (e.g., ranging from 0.03 to 0.91), which were observed in the dataset [27]. The GLMM approach accounts for both within-study and between-study variability without requiring continuity corrections, making it well-suited for the dataset’s characteristics [28]. The Maximum Likelihood (ML) estimator was used to estimate between-study variance (tau^2^) [29]. Confidence intervals for individual study proportions were constructed using the Wilson score method [30].

To explore differences in the prevalence of depression and anxiety between women with PCOS (treatment group) and controls (control group), a subgroup analysis was performed, with separate pooled estimates calculated for each subgroup [29]. Heterogeneity within and between subgroups was assessed using the I^2^ statistic and tau^2^ estimates to quantify the degree of variability [31]. The significance of heterogeneity was estimated using the Wald test and the Likelihood Ratio Test (LRT). The subgroup analysis was specified to allow distinct between-study variances for each subgroup and to avoid assuming a common effect across subgroups [29]. The back-transformation option was applied to present pooled proportions on the original scale for interpretability [26]. The difference between the proportions of depressive symptoms in the PCOS and control groups was assessed using a Wald-type test.

To visualize the pooled estimates, subgroup differences and heterogeneity statistics were analyzed using forest plots. They were reported with 95% confidence intervals. The presence of influential studies was checked using the “rules of thumb” by estimating externally standardized residual, DFFITS value, Cook’s distance, covariance ratio, the leave-one-out amount of (residual) heterogeneity, the leave-one-out test statistic of the test for (residual) heterogeneity and DFBETAS value(s) as described in Viechtbauer and Cheung [32]. To evaluate the potential for publication bias in the meta-analysis, a funnel plot was generated to visualize the distribution of study-specific proportion estimates against their precision (standard error) [33]. Asymmetry in the funnel plot, which may indicate publication bias or other small-study effects, was assessed visually and statistically. The Peters test was conducted to formally test for funnel plot asymmetry, as it is specifically designed for meta-analyses of proportions and accounts for the binomial nature of the data [34].

### 2.6. Characteristics of the Statistical Tools

Analyses were conducted using the R Statistical language version 4.3.3 [35] on Windows 11 Pro 64 (build 26100), using the packages meta version 7.0.0 [36], dmetar version 0.1.0 [37], report version 0.5.8 [38], and dplyr version 1.1.4 [39].

## 3. Results

### 3.1. Pooled Prevalence of Depressive Symptoms in Women with Polycystic Ovary Syndrome Compared to a Control Population

The meta-analysis aggregated data from 35 studies, spanning 2006 to 2024 and included 3610 women diagnosed with PCOS and 2247 control women, totaling 5857 participants across all studies [19,20,21,40,41,42,43,44,45,46,47,48,49,50,51,52,53,54,55,56,57,58,59,60,61,62,63,64,65,66,67,68,69,70,71]. The majority of studies (32) applied the Rotterdam criteria for PCOS diagnosis, while three used the National Institutes of Health (NIH) criteria, and three did not specify diagnostic criteria. The studies’ sample sizes ranged from 43 to 429 participants, with PCOS group sizes varying from 25 to 300 (median: 100) and control group sizes varying from 18 to 199 (median: 85).

The mean age of women with PCOS ranged from 21.6 to 35.9 years (median: 27.4 years). Control women had mean ages between 22.4 and 36.4 years (median: 29.3 years). Variability in age was moderate, with standard deviations or interquartile ranges typically between 0.9 and 10.4 years for PCOS participants and 1.1 to 10.4 years for controls.

BMI data were available in 26 studies for PCOS participants and 24 studies for controls. Mean BMI for women with PCOS ranged from 21.4 to 34.9 kg/m^2^ (median: 27.2 kg/m^2^), often indicating overweight or obese status. Control women had mean BMIs from 20.8 to 34.5 kg/m^2^ (median: 24.4 kg/m^2^). Variability in BMI was evident, with standard deviations or interquartile ranges such as 2.4–8.5 kg/m^2^ for PCOS and 0.8–6.3 kg/m^2^ for controls.

Depressive symptoms in this meta-analysis were evaluated using four standardized instruments: the BDI, applied in 17 studies; the HADS-D, used in 15 studies; the PRIME-MD PHQ, used in 2 studies; the PHQ-9, applied in 1 study; and the Mini-NPI, utilized in 3 studies.

#### 3.1.1. Pooled Prevalence of Depressive Symptoms Assessed by the BDI

**Figure 2:**
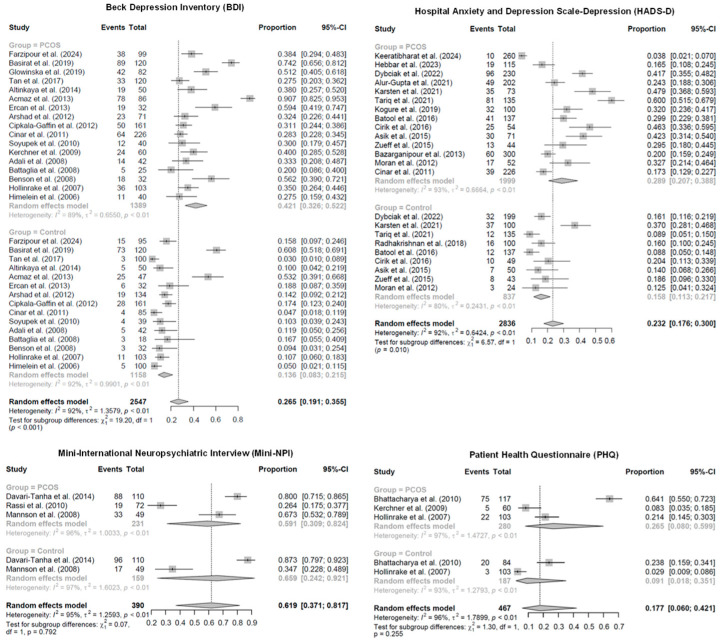
Forest plots of pooled prevalence of depressive symptoms assessed by the BDI, the HADS-D, the Mini-NPI and the PHQ in women with PCOS and controls, using a random-effects model [19,20,21,40,41,42,43,44,45,46,47,48,49,50,51,52,53,54,55,56,57,58,59,60,61,62,63,64,65,66,67,68,69,70,71].

The meta-analysis included 32 studies, comprising 2547 observations and 784 events of depressive symptoms assessed by the BDI tool [40,41,42,44,46,48,50,52,53,56,57,58,60,61,64,69,70] in the R statistical software [25]. No influential studies were detected, indicating that the results were robust and not driven by any single study. The overall pooled prevalence of depressive symptoms, estimated using a random-effects model, was 26.5% (95% CI: 19.1% to 35.5%) (Figure 2). Substantial heterogeneity was observed across studies, with a between-study variance of tau^2^ = 1.36, I^2^ = 92.1% (95% CI: 89.8% to 93.8%), and a heterogeneity test statistic of Q = 390.4 (df = 31, *p* < 0.001), indicating significant variability.

Subgroup analysis compared the prevalence of depressive symptoms between women with PCOS (treatment group, k = 17 studies) and controls (control group, k = 15 studies). The pooled prevalence was higher in the PCOS group at 42.1% (95% CI: 32.6% to 52.2%) compared to 13.6% (95% CI: 8.4% to 21.5%) in the control group (Figure 2). Heterogeneity remained high within subgroups, with tau^2^ = 0.66 (I^2^ = 89.4%, Q = 150.8) for the PCOS group and tau^2^ = 0.99 (I^2^ = 91.9%, Q = 173.6) for the control group. The test for subgroup differences was statistically significant (Q = 19.2, df = 1, *p* < 0.001), indicating a significant difference in depression prevalence between women with PCOS and controls.

**Figure 3:**
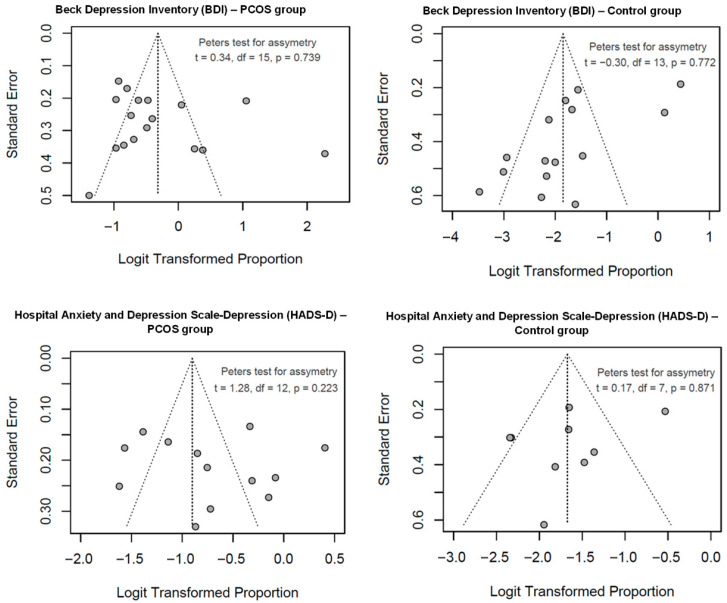
Funnel plots of logit-transformed proportions for depressive symptoms (BDI; HADS-D) in women with PCOS and controls, with Peters test for asymmetry indicating no significant asymmetry.

For the PCOS group, visual inspection of a funnel plot (Figure 3) revealed no obvious asymmetry, indicating no clear evidence of publication bias or small-study effects. The Peters test yielded a test statistic of t = 0.34 (df = 15, *p* = 0.739), with a bias estimate of 7.85 (standard error = 23.1), revealing no significant asymmetry and supporting the visual finding of no publication bias within the PCOS subgroup.

Visual inspection of the funnel plot for the control group (Figure 3) similarly showed no apparent asymmetry, consistent with the absence of small-study effects. The Peters test resulted in a test statistic of t = −0.30 (df = 13, *p* = 0.772), with a bias estimate of −10.0 (standard error = 33.8), further confirming no significant asymmetry in the control subgroup. The non-significant *p*-values in both subgroups suggest that publication bias is unlikely to have influenced the meta-analysis results. However, interpretation was cautious due to the moderate number of studies (17 for PCOS, 15 for control) and potential confounding from the high heterogeneity observed in the overall analysis.

In conclusion, the meta-analysis demonstrates a significant positive correlation between PCOS and depression scores on the BDI assessment tool. Women with PCOS exhibited a markedly higher pooled prevalence of depressive symptoms (42.11%) compared to controls (13.62%), with a statistically significant subgroup difference (*p* < 0.001). These findings highlight the increased burden of depression in women with PCOS, underscoring the need for targeted mental health interventions in this population.

#### 3.1.2. Pooled Prevalence of Depressive Symptoms Assessed by the HADS-D

The meta-analysis included 23 studies, comprising 2836 observations and 684 events of depressive symptoms assessed by the HADS-D [19,20,21,43,45,47,49,52,54,59,62,63,66,67,71]. The overall pooled prevalence of depressive symptoms, estimated using a random-effects model, was 23.23% (95% CI: 17.62% to 29.96%) (Figure 2). Substantial heterogeneity was observed across studies, with a between-study variance of tau^2^ = 0.64, I^2^ = 91.8% (95% CI: 88.9% to 93.9%), and a heterogeneity test statistic of Q = 267.0 (df = 22, *p* < 0.001).

Subgroup analysis compared the prevalence of depressive symptoms between women with PCOS (treatment group, k = 14 studies) and controls (control group, k = 9 studies). The pooled prevalence was higher in the PCOS group at 28.9% (95% CI: 20.7% to 38.8%) compared to 15.8% (95% CI: 11.3% to 21.7%) in the control group (Figure 2). Heterogeneity remained high within subgroups, with tau^2^ = 0.67 (I^2^ = 93.0%, Q = 185.3) for the PCOS group and tau^2^ = 0.24 (I^2^ = 79.6%, Q = 39.2) for the control group. The test for subgroup differences was statistically significant (Q = 6.6, df = 1, *p* = 0.010), indicating a significant difference between the proportions of depressive symptoms in women with PCOS and controls.

A funnel plot for the PCOS subgroup, presented in Figure 3, showed no asymmetry. The Peters test provided a test statistic of t = 1.28 (df = 12, *p* = 0.224), with a bias estimate of 49.9 (standard error = 38.9). For the control subgroup, the funnel plot in Figure 3 also lacked asymmetry upon visual inspection. The Peters test yielded a test statistic of t = 0.17 (df = 7, *p* = 0.872), with a bias estimate of 5.1 (standard error = 30.7). The non-significant *p*-values (*p* = 0.224 for PCOS, *p* = 0.872 for control) indicated no evidence of publication bias.

As a conclusion, the analysis revealed a higher prevalence of depressive symptoms among women with PCOS (28.9%) compared to the control group (15.8%), with a statistically significant difference (*p* = 0.010). This indicates an elevated risk of depression in women with PCOS, warranting clinical attention to screening and management of depressive symptoms in this population.

#### 3.1.3. Pooled Prevalence of Depressive Symptoms Assessed by the Mini-NPI

The meta-analysis included 5 studies, comprising 390 observations and 253 events of depressive symptoms assessed by the Mini-NPI [55,65,68]. The overall pooled prevalence of depressive symptoms, estimated using a random-effects model, was 61.9% (95% CI: 37.1% to 81.7%) (Figure 2). Substantial heterogeneity was observed across studies, with a between-study variance of tau^2^ = 1.26, I^2^ = 95.3% (95% CI: 91.7% to 97.4%), and a heterogeneity test statistic of Q = 86.0 (df = 4, *p* < 0.001).

Subgroup analysis compared the prevalence of depressive symptoms between women with PCOS (treatment group, k = 3 studies) and controls (control group, k = 2 studies). The pooled prevalence was 59.1% (95% CI: 30.9% to 82.4%) in the PCOS group and 65.9% (95% CI: 24.2% to 92.1%) in the control group (Figure 2). Heterogeneity remained high within subgroups, with tau^2^ = 1.0 (I^2^ = 95.7%, Q = 46.7) for the PCOS group and tau^2^ = 1.60 (I^2^ = 97.4%, Q = 38.1) for the control group. The test for subgroup differences was not statistically significant (Q = 0.07, df = 1, *p* = 0.792), indicating no significant difference between the proportions of depressive symptoms in women with PCOS and controls.

#### 3.1.4. Pooled Prevalence of Depressive Symptoms Assessed by the PHQ

The meta-analysis included five studies, comprising 467 observations and 125 events of depressive symptoms assessed by the PHQ [51,61,64]. The overall pooled prevalence of depressive symptoms, estimated using a random-effects model, was 17.74% (95% CI: 6.0% to 42.1%) (Figure 2). Substantial heterogeneity was observed across studies, with a between-study variance of tau^2^ = 1.79, I^2^ = 95.5% (95% CI: 92.1% to 97.5%), and a heterogeneity test statistic of Q = 89.8 (df = 4, *p* < 0.001).

Subgroup analysis compared the prevalence of depressive symptoms between women with PCOS (treatment group, k = 3 studies) and controls (control group, k = 2 studies). The pooled prevalence was 26.5% (95% CI: 8.0% to 59.9%) in the PCOS group and 9.1% (95% CI: 1.8% to 35.1%) in the control group (Figure 2). Heterogeneity remained high within subgroups, with tau^2^ = 1.47 (I^2^ = 96.6%, Q = 58.2) for the PCOS group and tau^2^ = 1.28 (I^2^ = 92.6%, Q = 13.4) for the control group. The test for subgroup differences was not statistically significant (Q = 1.30, df = 1, *p* = 0.255), indicating no significant difference between the proportions of depressive symptoms in women with PCOS and controls.

### 3.2. Pooled Prevalence of Anxiety Symptoms in Women with Polycystic Ovary Syndrome Compared to a Control Population

The meta-analysis aggregated data from 15 studies conducted between 2011 and 2024, encompassing 2099 women diagnosed with PCOS and 931 control women, totaling 3030 participants [19,20,21,45,47,49,52,54,59,62,63,66,67,71]. The majority of studies (13) utilized the Rotterdam criteria for PCOS diagnosis, and two did not specify diagnostic criteria. Study sample sizes ranged from 43 to 429 participants, with PCOS group sizes varying from 44 to 300 (median: 100) and control group sizes varying from 24 to 199 (median: 93).

The mean age with PCOS ranged from 22.0 to 34.1 years (median: 28.0 years), with variability indicated by standard deviations or interquartile ranges typically between 1.3 and 8.6 years. Control women had mean ages between 24.0 and 36.4 years (median: 29.0 years), with variability ranging from 1.7 to 8.3 years. BMI data were available in 10 studies for PCOS participants and 8 for controls. The mean BMI for women with PCOS ranged from 24.7 to 34.6 kg/m^2^ (median: 30.1 kg/m^2^), often indicating an overweight status, with variability from 2.3 to 7.1 kg/m^2^. Control women exhibited mean BMIs from 21.0 to 34.5 kg/m^2^ (median: 26.1 kg/m^2^), with variability between 0.8 and 5.4 kg/m^2^.

#### Pooled Prevalence of Anxiety Symptoms Assessed by the HADS-A

**Figure 4:**
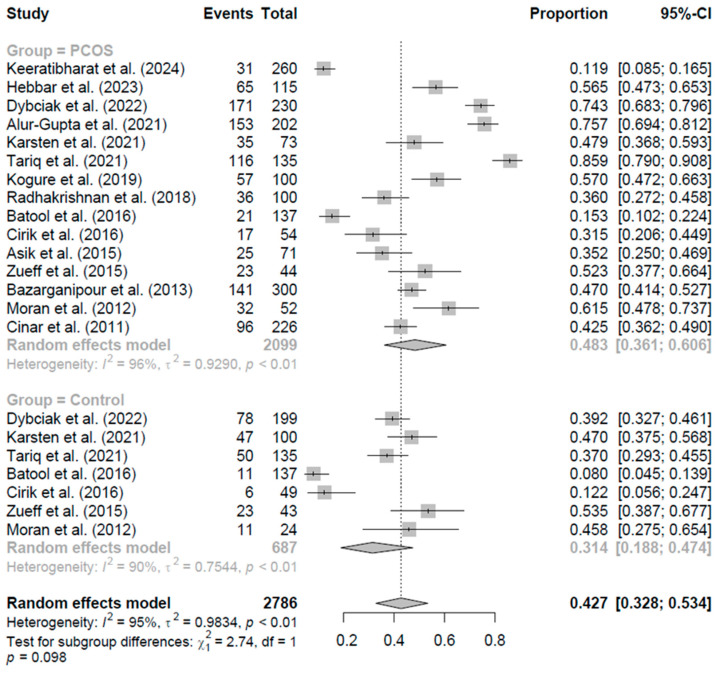
Forest plot of pooled prevalence of anxiety symptoms assessed by the HADS-A in women with PCOS and controls, using a random-effects model [19,20,21,45,47,49,52,54,59,62,63,66,67,71].

The meta-analysis included 22 studies, comprising 2786 observations and 1245 events of anxiety symptoms assessed by the HADS-A [19,20,21,45,47,49,52,54,59,62,63,66,67,71]. The overall pooled prevalence of anxiety symptoms, estimated using a random-effects model, was 42.8% (95% CI: 32.8% to 53.4%) (Figure 4). Substantial heterogeneity was observed across studies, with a between-study variance of tau^2^ = 0.98, I^2^ = 95.2% (95% CI: 93.7% to 96.3%), and a heterogeneity test statistic of Q = 433.8 (df = 21, *p* < 0.001).

Subgroup analysis compared the prevalence of anxiety symptoms between women with PCOS (treatment group, k = 15 studies) and controls (control group, k = 7 studies). The pooled prevalence was 48.25% (95% CI: 36.1% to 60.6%) in the PCOS group and 31.4% (95% CI: 18.8% to 47.4%) in the control group (Figure 4). Heterogeneity remained high within subgroups, with tau^2^ = 0.93 (I^2^ = 96.0%, Q = 346.0) for the PCOS group and tau^2^ = 0.75 (I^2^ = 89.6%, Q = 57.9) for the control group. The test for subgroup differences was not statistically significant (Q = 2.74, df = 1, *p* = 0.098), indicating no significant difference between the proportions of anxiety symptoms in women with PCOS and controls.

**Figure 5:**
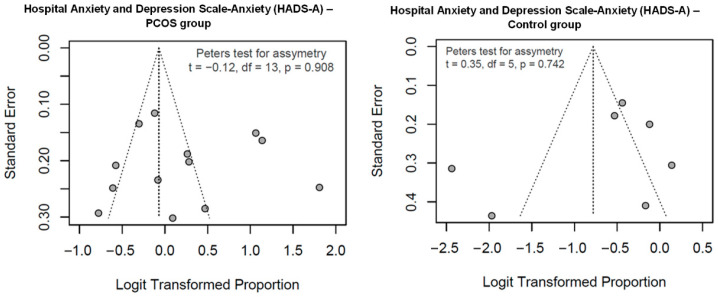
Funnel plot of logit-transformed proportions for anxiety symptoms (HADS-A) in women with PCOS and controls, with Peters test for asymmetry indicating no significant asymmetry.

Visual inspection of the funnel plot for the PCOS group (Figure 5) showed no apparent asymmetry, revealing the absence of small-study effects. The Peters test for funnel plot asymmetry yielded a test statistic of t = −0.12 (df = 13, *p* = 0.908), with a bias estimate of −5.97 (standard error = 50.92), indicating no significant asymmetry in the PCOS subgroup. Similarly, the funnel plot for the control group (Figure 5) revealed no clear asymmetry, consistent with minimal small-study effects. The Peters test for the control group resulted in a test statistic of t = 0.35 (df = 5, *p* = 0.742), with a bias estimate of 11.77 (standard error = 33.83), further supporting the absence of significant asymmetry. The non-significant *p*-values in both subgroups indicate that publication bias is unlikely to have influenced the meta-analysis results. However, interpretation was cautious due to the small number of studies (*n* = 7 for control) and potential confounding from the high heterogeneity observed in the overall analysis.

The analysis revealed a higher prevalence of anxiety symptoms among women with PCOS (48.25%) compared to the control group (31.40%), though the difference was not statistically significant (*p* = 0.098). This infers a potential elevated risk of anxiety in women with PCOS, warranting clinical consideration for screening and management of anxiety symptoms in this population.

## 4. Discussion

The number of scientific reports suggesting that women with PCOS may be at higher risk of depression, anxiety, bipolar disorder, obsessive–compulsive disorder, and sleep disorders compared to the general population is constantly increasing [10,11,12]. Hollinrake et al. (2007) revealed that depression and anxiety are the most common mental disorders in women with PCOS [61]. The aim of this meta-analysis was to determine the prevalence of depressive and anxiety symptoms in women with polycystic ovary syndrome compared to the general adult female population. For this purpose, an analysis was performed on a group of 5857 women (study group: 3610; control group: 2247) from 35 studies that met the inclusion criteria.

In our study, we found that women with PCOS had significantly higher levels of depressive symptoms compared to women without PCOS, which has been previously stated by other researchers [8,17,72]. It has been proven that the overall probability of depression in patients with PCOS is more than 2.5-fold higher than in healthy women [8], and patients with PCOS are not only at increased risk of developing any symptoms of depression or anxiety, but also moderate to severe symptoms [17]. Our results confirm these estimates, indicating that the risk of developing depression or anxiety among PCOS patients may be more than 3-fold higher and 1.5-fold higher, respectively, than in healthy women. These findings emphasize that both disorders should be considered a major concern in the multidisciplinary care of PCOS patients.

In addition, the presence of acne, high levels of perceived stress, and evening chronotype, as well as lack of regular physical activity and a high-fat diet, were identified as significant risk factors for the co-occurrence of depression and PCOS [72]. However, the mechanisms underlying the simultaneous development of these conditions are not yet entirely understood, although a multifactorial background has been suggested [17,19,20,21]. Women with PCOS experience disruptive symptoms such as hirsutism, acne, infertility, and abdominal obesity, which can result in body image dissatisfaction, social withdrawal, and low self-esteem [63]. These implications can account for higher levels of perceived stress and greater emotional distress than in the general population, which may further contribute to the development of depressive symptoms [41]. The role of stress in the origin of depression may be conceptualized as the result of multiple converging factors, all of which have the capacity to induce persistent hyperactivity of the hypothalamic–pituitary–adrenal (HPA) axis. These changes, which include increased availability of corticotropin-releasing factor and cortisol, are also associated with hyperactivity of the amygdala, hypoactivity of the hippocampus, and decreased serotonergic neurotransmission. Collectively, these factors lead to increased vulnerability to stress [73]. In PCOS, this can result in a vicious cycle in which distress has a detrimental effect on the individual’s body image, and conversely, a negative body image can exacerbate feelings of distress. The aforementioned factors provide a comprehensive rationale for the conceptualization of a bidirectional link between stress and the potential for depressive symptoms to emerge [12,74,75]. Metabolic disorders, including insulin resistance, dyslipidaemia, hypertriglyceridaemia, and reduced HDL cholesterol levels, also remain a relevant issue in terms of mental disorders in PCOS [9]. As demonstrated conclusively by numerous studies, higher levels of fasting glucose, fasting insulin, an insulin resistance index (HOMA-IR), and lipid parameters, as well as the free androgen index (FAI), have been shown to be positively correlated with the occurrence of depressive symptoms [42,45,47,49,61]. In recent years, research on lipid metabolism parameters concerning free fatty acids has become prominent. It has been proven that high myristic acid (C14:0), palmitoleic acid (C16:1), oleic acid (C18:1n-9C), cis-vaccenic acid (C18:1n-7), and homo-gamma-linolenic acid (C20:3n-6) levels are associated with metabolic risk in women with PCOS, independent of BMI, and these fatty acids have emerged as potential biomarkers [76]. Subsequently, high concentrations of free fatty acids lead to increased production of inflammatory markers, which, after crossing the blood–brain barrier, may promote the development of depression [77]. In comparison with the general population, depressed individuals have been shown to exhibit elevated circulating levels of several proinflammatory cytokines, including IL-1, IL-6, and TNF-α, as well as higher levels of the systemic inflammatory biomarker, C-reactive protein (CRP) [78,79]. Despite their relative size, which hinders passive transport across the blood–brain barrier, cytokines have the capacity to modify neural activity via at least three distinct pathways, encompassing cellular, molecular, and neural mechanisms. Firstly, circulating cytokines can enter the central nervous system in areas where the blood–brain barrier is either incomplete (e.g., circumventricular sites) or permeable (e.g., organum vasculosum of the lamina terminalis). Secondly, peripheral cytokines are able to communicate with the brain by binding to the cerebral vascular endothelium, thereby facilitating the release of second messengers and the induction of local cytokine activity within the brain. Finally, carrier-mediated mechanisms have been demonstrated to facilitate the active transport of cytokines across the blood–brain barrier [80]. The association between inflammation and depression may be elucidated by the process of cell-mediated immune activation, which encompasses the functions of macrophages, monocytes, and T-lymphocytes. Individuals diagnosed with depression have been shown to present with increased numbers of T cells, including CD4+ (T helper cells) and CD8+ (T suppressor/cytotoxic cells), as well as elevated levels of the soluble receptors for IL-2 and TNF-α. It has been hypothesized that these imbalances, in conjunction with increased cytokine activity, may result in a reduction in serotonin levels by decreasing the availability of its precursor, an amino acid termed tryptophan. This, in turn, may lead to the exacerbation of somatic and neurovegetative depressive symptoms [77,79]. Additionally, insulin resistance has been demonstrated to promote the onset of mental symptoms by instigating inflammatory responses and modulating the HPA axis [81,82,83]. The reduced activity of lecithin–cholesterol acyltransferase (LCAT), an enzyme secreted by the liver with a preferential affinity for free cholesterol contained in HDL particles, is also raised as a potential risk factor for the onset of major depressive disorder [84]. This particular pathology has been observed to be accompanied by increased levels of apolipoprotein E (ApoE), the predominant apolipoprotein of chylomicrons, which is essential for the degradation of triglyceride-rich lipoprotein components. The ApoE protein is predominantly expressed in liver cells, but also in gonadal cells, macrophages, and astrocytes within the brain. Over the years, there has been a great deal of speculation concerning the hypothesis that ApoE gene polymorphism status may serve as a risk factor for depression. The ApoE ε4 allele was a particular focus of the study, due to its recognized propensity to promote inflammatory and neurotoxic processes. However, recent findings have revealed that the ApoE ε4 allele increases the risk of depression, and depressive patients who carry the ApoE ε4 allele present with more severe depressive symptoms [84,85,86]. This finding provides an opportunity for earlier detection of susceptibility to the disorder. However, obesity remains one of the most well-documented risk factors for depression, in the general population as well [87]. Of particular concern are alterations that may provide a rationale for homeostatic regulation of this bidirectional link, including immune-inflammatory activation, gut microbiota, neuroplasticity, HPA axis dysregulation, and neuroendocrine regulators of metabolism, such as adipocytokines and lipokines. The prevailing directions for investigating the pathogenesis of depression currently include gut microbiota and neuroplasticity, while the secretion of adipokines (e.g., leptin, adiponectin, visfatin) and lipokines (e.g., palmitoleic acid, lysophosphatidic acid) plays a crucial role in all of these pathways. Gut microbiota disturbances observed in the co-pathogenesis of obesity and depression primarily refer to a decreased number of short-chain fatty acid (SCFA)-producing bacteria, such as Roseburia, Ruminococcus and Eubacterium. A decrease in the amount of gut microbiota metabolites results in dysregulation of the gut–brain axis (GBA) via vagal stimulation, and abnormal central appetite signalling, a phenomenon that is a critical factor in the development of obesity. Another pathway pertains to the concept of neuroplasticity, which is defined as the brain’s capacity to undergo neurobiological alterations in response to external stimuli. The hypothesis that advanced brain functions require synaptic frequency decoding is predicated on the premise that alterations in synaptic activation frequency can engender an augmentation or diminution in the long-term efficiency of these synapses, which may consequently precipitate long-term potentiation (LTP) or long-term depression (LTD). Recent neuroscience research has indicated a correlation between depressive-like behaviours and chronic stress, with damage to neuroplasticity. In depressed individuals, a decline in TLP was observed, accompanied by an increase in TLD, as well as neuronal atrophy and synaptic loss in the medial prefrontal cortex and hippocampus. Furthermore, it has been observed that chronic sugar consumption has a detrimental effect on neuroplasticity, reducing impulse control and, consequently, resistance to high-fat and high-sugar products. However, the neural mechanism through which obesity and depression interact remains largely inscrutable [87,88,89,90,91,92,93]. In our study, women with PCOS had higher average BMI values than healthy women, often indicating overweight or obese status (median: 27.2 kg/m^2^ vs. 24.4 kg/m^2^), which is consistent with the results obtained by other researchers [40,41,42,43,53,61,94]. Considering the associations between hormonal activity of adipose tissue, clinical hyperandrogenism and metabolic disorders, as well as psychological burdens, including body dissatisfaction, modification of these factor should be implemented in the treatment of PCOS-related depression [17,21,95]. Contrary to prevailing assumptions, emerging evidence indicates that in cases of concurrent depression and obesity, it is advantageous to prioritize the treatment of depression. The efficacy of cognitive behavioural therapies in addressing co-occurring depression and obesity has been well-documented. In addition, preliminary evidence suggests that enhancing emotion regulation and reducing weight bias internalization may be significant treatment targets [96].

In addition to investigating the prevalence of depressive symptoms, the study analyzed the prevalence of anxiety symptoms in women with PCOS. Following the HADS-A results, we observed a tendency towards a higher incidence of anxiety symptoms in the PCOS group, although the difference did not reach statistical significance in the pooled analysis. This non-significant outcome may be due to the high heterogeneity among the few studies on anxiety symptoms that were included in the analysis. However, publication bias was unlikely to have affected the meta-analysis results, and the observed finding is consistent with numerous studies suggesting that anxiety is more prevalent in women with PCOS than in the general population [11,17,82,97].

The mechanisms linking PCOS to anxiety symptoms are thought to largely coincide with those associated with depression. The main risk factors included high levels of perceived stress, depressive symptoms, and intolerance of uncertainty, which refers to an individual’s inability to cope with ambiguous or unpredictable situations [82]. In the preceding two decades, human functional imaging has identified multiple cerebral regions, including the hypothalamus, basolateral amygdala, medial prefrontal cortex and nuclei, which are active during both stress and anxiety responses. The intermingled neural circuits responsible for regulating these behaviours suggest a robust bidirectional relationship between stress experiences and anxiety in both healthy and pathological conditions. Consequently, alterations in the connectivity between the aforementioned brain regions may contribute to the etiology of psychopathologies such as generalized anxiety disorder (GAD), social anxiety disorders or post-traumatic stress disorder (PTSD) [98]. A higher incidence of clinical symptoms such as weight gain, acne, hirsutism, and androgenetic alopecia, as well as higher levels of testosterone, dehydroepiandrosterone sulphate (DHEA-S), and luteinizing hormone (LH) to follicle-stimulating hormone (FSH) ratio, were also identified [82,99]. The extant research suggests that elevated testosterone levels have the potential to exacerbate anxiety symptoms by exerting an influence on the activity of emotional processing centres, including the amygdala [82]. Furthermore, a positive correlation was observed between elevated levels of HOMA-IR, altered lipid parameters, and increased FAI, and the occurrence of anxiety symptoms [42,49]. These abnormal hormone levels may be related to excessive adipose tissue accumulation, and in our analysis, we observed that PCOS patients had higher average BMI values, often indicating overweight status (median: 30.1 kg/m^2^ vs. 26.1 kg/m^2^), than healthy women. A similar association has been observed in previous research [99,100]. Finally, women with PCOS experience profound uncertainty and anxiety about their reproductive health. Despite the absence of statistically significant differences in the sexual function of women with PCOS when compared to the general population [21,46,48,56,59,71], a negative correlation was identified between the overall Female Sexual Function Index (FSFI) score and the occurrence of anxiety symptoms [21]. The correlation was also observed in three subgroups of the questionnaire: desire, arousal, and lubrication. A more pronounced negative correlation was observed for the overall FSFI score and symptoms of depression, as well as for five subgroups of the questionnaire: desire, arousal, lubrication, orgasm, and pain [21,56]. This finding may serve as an additional indication of the integrality of the development of depressive and anxiety symptoms in PCOS. Moreover, sexual dysfunction was correlated with elevated concentrations of total testosterone, free testosterone, LH, and fasting insulin, which are recognized as characteristic endocrine abnormalities in PCOS [56]. In addition to the above, imbalances in sex hormone-binding globulin (SHBG), androstenedione, and progesterone concentrations were identified among women diagnosed with PCOS, and these abnormalities may contribute to ovulation disorders [48,66]. The aforementioned dysfunctions generate substantial psychological distress, but also affect women’s mood, self-esteem, and social functioning [82]. Undoubtedly, PCOS is embedded within a sociocultural framework where femininity, fertility, and appearance are subject to social scrutiny. This phenomenon is of particular concern for women navigating unstable social or intimate relationships, as external pressures can exacerbate underlying psychological conditions, including anxiety [101]. Furthermore, delays in the diagnosis of PCOS have the potential to result in the loss of opportunities for symptom management, the provision of counselling on fertility, and the initiation of preventive strategies for metabolic complications. This underscores the significance of addressing psychological aspects in a timely manner [102]. Due to the multifaceted nature of disorders co-occurring with PCOS, the syndrome cannot be treated as one-dimensional and remains a therapeutic challenge for physicians of multiple specializations.

The present meta-analysis has several strengths. The study encompassed recent research findings identified up to May 2025 in three prominent and widely recognized research databases, in which the PCOS diagnosis was confirmed by a specialist, as opposed to being self-reported by the patients. The studies incorporated into the analysis utilized standardized questionnaires, which have been documented as screening tools with a high degree of accuracy. This ensured the consistency and efficacy of the statistical analysis outcomes. Furthermore, the research contemplated the occurrence of sexual dysfunction and hormonal correlations with depressive and anxiety symptoms in PCOS, as well as mechanisms potentially underlying the pathogenesis of depression and anxiety disorders. Moreover, the study provides extensive Supplementary Material, which may serve as a valuable source of data for other researchers. Nevertheless, the current study is not without its limitations. Firstly, the protocol had not been submitted to the PROSPERO registry, as the data extraction had been initiated prior to the decision to formally register the study. However, procedural transparency remained uncompromised, and the analysis was conducted in accordance with the PRISMA guidelines. In light of the prevailing standards, a decision was also taken to incorporate prior registration in the PROSPERO database for subsequent projects at the planning stage. Secondly, statistical analysis excluded adolescent or post-menopausal women, resulting in a paucity of data concerning the association of PCOS and depressive or anxiety symptoms in those age groups. Thirdly, the subtypes of PCOS were not considered due to an absence of data in the published studies. Finally, the broad methodological range of the studies included in the anxiety symptoms analysis resulted in the inability to reach definitive conclusions, as evidenced by the heterogeneity of the sample size (I^2^ > 90% in several analyses). The presence of variability and heterogeneity in the characteristics under study has the potential to render statistical inference challenging and to exert a subtle influence on the conclusions of the analysis. The fact that data on the prevalence of depressive symptoms were collected using a variety of research tools is also worthy of note. The heterogeneity of results between the BDI, HADS-D, Mini-NPI, and PHQ stems primarily from differences in their theoretical constructs, the range of symptoms assessed, and the psychometric properties of each instrument. The BDI and PHQ-9 encompass a more extensive range of symptoms, incorporating somatic components, while the HADS-D deliberately omits them, which may result in underestimation of depression severity. The Mini-NPI is a tool designed to assess depressive symptoms in the context of neuropsychiatric disorders, thus offering a more nuanced perspective on the results. Nevertheless, it can be posited that a significant proportion of the analyzed bibliography evinces an association between the three pathologies, although in certain instances, the significance of this association remains indeterminate.

## 5. Conclusions

The meta-analysis demonstrates that polycystic ovary syndrome significantly increases the prevalence of depressive symptoms. PCOS patients may also be at greater risk of developing anxiety symptoms, according to the observed trend, although the study did not provide conclusive evidence on this issue. Considering the findings, we suggest that patients with PCOS should undergo psychiatric screening in order to identify concerning symptoms as early as possible and implement appropriate treatment. Due to the limited number and quality of included studies, these conclusions require further confirmation by larger-sample, multi-centre clinical trials.

## Figures and Tables

**Table 1:** The population, intervention, comparison, outcomes and study design (PICOS) strategy for the study.

PICOS	Application of the Criteria in the Study
Population	Adult women diagnosed with polycystic ovary syndrome
Intervention	None
Comparison	Studies with or without control group
Outcomes	Group size, prevalence of symptoms of depression and anxiety
Study design	Case–control, cross-sectional, prospective and retrospective studies written in English

## Data Availability

The data supporting the findings of this study are available from the corresponding author upon reasonable request.

## Supplementary Materials

The following supporting information can be downloaded at: https://www.mdpi.com/article/10.3390/jcm15103582/s1, Table S1: The PRISMA 2020 checklist. Table S2: Characteristics of the studies included in the analysis. Table S3: Probability of depression or anxiety disorder in PCOS patients.

## Abbreviations

The following abbreviations are used in this manuscript:

AMH	Anti-Müllerian Hormone
ApoE	apolipoprotein E
ASRM	The American Society for Reproductive Medicine
BAI	Beck Anxiety Inventory
BDI	Beck Depression Inventory
BMI	body mass index
CRP	c-reactive protein
DHEA-S	dehydroepiandrosterone sulphate
FAI	free androgen index
FSFI	Female Sexual Function Index
FSH	follicle-stimulating hormone
GAD	generalized anxiety disorder
GBA	gut–brain axis
GLMM	Generalized Linear Mixed Model
HA	hyperandrogenism
HADS	Hospital Anxiety and Depression Scale
HOMA-IR	homeostatic model assessment for insulin resistance
HPA	hypothalamic–pituitary–adrenal axis
LCAT	lecithin–cholesterol acyltransferase
LH	luteinizing hormone
LRT	Likelihood Ratio Test
LSAS	Liebowitz Social Anxiety Scale
TLD	long-term depression
LTP	long-term potentiation
Mini-NPI	Mini International Neuropsychiatric Interview
ML	Maximum Likelihood
NIH	The National Institutes of Health
PCOS	polycystic ovary syndrome
PHQ-9	Primary Care Evaluation of Mental Disorders Patient Health Questionnaire 9
PI3K	phosphoinositide kinase
PRIME-MD PHQ	Primary Care Evaluation of Mental Disorders Patient Health Questionnaire
PTSD	post-traumatic stress disorder
SCFA	short-chain fatty acid
SHBG	sex hormone-binding globulin
STAI	State-Trait Anxiety Inventory
