# Supplementary material for: Depressive and Anxiety Symptoms in Women with Polycystic Ovary Syndrome: A Meta-Analysis
Source: J Clin Med. 2026 May 7;15(10):3582. doi: 10.3390/jcm15103582 (PMC13207832; doi:10.3390/jcm15103582)
Supplement: Supplementary file 1 [file jcm-15-03582-s001.zip › Supplementary material - Tables S2, S3.pdf]

Table S2. Characteristics of the studies included in the analysis.

Study (year), country	PCOS diagnostic criteria	Variables used to match PCOS subjects and controls	Sample size		Age [year] (mean ± SD)		BMI [kg/m^2] (mean ± SD)	
			PCOS	Control	PCOS	Control	PCOS	Control
Açmaz et al. (2013), Turkey	Rotterdam	None	86<br>Hirsutism-acnea group: 35<br>Infertility group: 22<br>Obesity group: 29	47	25.60 ± 5.50<br>26.14 ± 4.98<br>24.32 ± 4.59<br>26.00 ± 6.58	27.77 ± 6.49	27.50 ± 5.20<br>24.45 ± 2.75<br>24.35 ± 3.48<br>33.59 ± 2.61	23.37 ± 3.13
Adali et al. (2008), Turkey	Rotterdam	Age	42	42	23.54 ± 3.13	24.45 ± 2.47	28.42 ± 4.30	24.11 ± 4.14
Altinkaya et al. (2014), Turkey	Rotterdam	Age	50	50	23.70 ± 5.30	24.40 ± 5.70	25.10 ± 3.70	22.10 ± 2.70
Alur-Gupta et al. (2021), USA	Rotterdam	None	272<br>White: 202<br>Black: 70	295<br>White: 109<br>Black: 186	Not stated<br>28.70 (IQR: 24.5–32.2)<br>29.20 (IQR: 25.2–32.2)	Not stated<br>32.60 (IQR: 27.9–40.7)<br>31.60 (IQR: 26.5–39.3)	Not stated<br>31.10 (IQR: 25.5–38.4)<br>36.50 (IQR: 31.0–41.8)	Not stated<br>25.20 (IQR: 22.3–31.5)<br>31.80 (IQR: 26.1–39.3)
Arshad et al. (2012), Iran	Rotterdam	None	71	134	25.50 ± 5.60	29.60 ± 6.70	27.20 ± 5.70	24.90 ± 4.80
Asik et al. (2015), Turkey	Rotterdam	Age	71	50	22.00 (IQR: 18.0–32.0)	24.00 (IQR: 18.0–37.0)	27.00 (IQR: 17.0–47.0)	21.00 (IQR: 16.0– 37.0)
Basirat et al. (2019), Iran	Rotterdam	Age, level of education, duration of infertility	120	120	29.55 ± 5.17	29.33 ± 6.23	Not stated	Not stated
Batool et al. (2016), Pakistan	Not stated	None	137	137	25.00 ± 8.60	26.00 ± 8.30	33.60 ± 7.10	27.00 ± 5.30
Battaglia et al. (2008), Italy	NIH	Age, BMI, Caucasian	25	18	27.70 ± 5.40	30.70 ± 3.90	21.60 ± 2.40	21.20 ± 2.00

Bazarganipour et al. (2013), Iran	Rotterdam	–	300	–	26.56 ± 4.44	–	Not stated	–
Benson et al. (2008), Germany	NIH	BMI	32	32	30.10 ± 0.90	31.50 ± 1.10	29.80 ± 1.60	28.70 ± 1.50
Bhattacharya et al. (2010), India	Rotterdam	None	117	84	21.60 ± 3.40	22.40 ± 20	26.20 ± 4.10	24.50 ± 3.50
Cinar et al. (2011), Turkey	Rotterdam	BMI	226	85	23.20 ± 5.20	24.40 ± 4.00	24.70 ± 5.70	23.40 ± 5.40
Cipkala-Gaffin et al. (2012), USA	Not stated	Age, ethnicity, demographics	161	161	35.20 ± 7.30	36.40 ± 7.80	30.30 ± 8.20	26.30 ± 6.30
Cirik et al. (2016), Turkey	Rotterdam	None	101<br>NIH-phenotype: 54<br>Non-NIH-phenotype: 47	49	Not stated<br>24.70 ± 4.39<br><br>24.15 ± 4.08	26.29 ± 5.17	Not stated<br>25.84 ± 4.81<br><br>23.88 ± 8.45	24.44 ± 3.88
Davari-Tanha et al. (2014), Iran	Rotterdam	Age, infertility	110	110	29.59 ± 5.60	30.99 ± 7.30	30.54 ± 4.10	29.03 ± 3.40
Dybciak et al. (2022), Poland	Rotterdam	Age	230	199	Not stated	Not stated	Not stated	Not stated
Ercan et al. (2013), Turkey	Rotterdam	Age	32	32	27.40 ± 3.30	27.00 ± 3.20	25.50 ± 3.00	24.40 ± 3.6
Farzipour et al. (2024), Iran	Rotterdam	Age	99	95	Not stated	Not stated	Not stated	Not stated
Glowinska et al. (2019), Poland	Rotterdam	–	82	–	28.00 ± 5.48	–	26.00 ± 5.30	–
Hebbar et al. (2023), UK, India	Rotterdam	–	115<br>UK: 36<br>India: 79	–	Not stated	–	Not stated	–
Himelein et al. (2006), USA	Rotterdam	None	40	100	32.10 ± 5.50 (both groups)		34.00 ± 9.00	23.00 ± 4.00
Hollinrake et al. (2007), USA	Rotterdam	None	103	103	29.80 ± 6.20	30.70 ± 8.50	34.90 ± 8.50	25.40 ± 4.70

Karsten et al. (2021), Netherlands	Rotterdam	BMI, infertility	73	100	34.10 ± 4.10	35.10 ± 3.70	34.60 ± 5.30	34.40 ± 4.90
Keeratibharat et al. (2024), Thailand	Rotterdam	–	260	–	28.00 ± 4.90	–	26.20 ± 6.40	–
Kerchner et al. (2009), USA	Rotterdam	–	60	–	32.00 ± 6.00	–	33.90 ± 8.30	–
Kogure et al. (2019), Brazil	Rotterdam	–	100	–	28.80 ± 5.10	–	29.10 ± 5.30	–
Mannson et al. (2008), Sweden	Rotterdam	Age	49	49	35.90 ± 10.40	35.90 ± 10.40	29.00 ± 7.00	23.50 ± 3.00
Moran et al. (2012), Australia	Rotterdam	None	52	24	32.60 ± 1.30	36.40 ± 1.70	34.40 ± 2.30	28.70 ± 0.80
Radhakrishnan et al. (2018), India	Not stated	–	100	–	Not stated	–	Not stated	–
Rassi et al. (2010), Brazil	NIH	–	72	–	26.20 ± 5.05	–	Not stated	–
Soyupek et al. (2010), Turkey	Rotterdam	Age	40	39	24.10 ± 6.13	26.14 ± 5.67	24.17 ± 5.60	21.81 ± 3.82
Tan et al. (2017), China	Rotterdam	None	120	100	24.80 ± 3.80	25.00 ± 3.50	21.40 ± 3.00	20.80 ± 1.90
Tariq et al. (2021), Pakistan	Rotterdam	Age, BMI, demographics	135	135	Not stated	Not stated	Not stated Height [m]: 162.10 ± 6.70 Weight [kg]: 93.30 ± 40.30	Not stated Height [m]: 156.40 ± 3.76 Weight [kg]: 58.20 ± 6.90
Zueff et al. (2015), Brasil	Rotterdam	BMI	44	43	30.10 ± 4.90	32.60 ± 4.60	34.50 ± 2.90	34.50 ± 3.00

Note. BMI – Body Mass Index; IQR – the interquartile range; NIH – The National Institutes of Health; PCOS – polycystic ovary syndrome; UK – United Kingdom; USA – United States of America.

Table S3. Probability of depression or anxiety disorder in PCOS patients.

Study (year), country	Depression screening tool	Anxiety screening tool	Med exclusion	Depressive symptoms (mean ± SD) [%]		Anxiety (mean ± SD) [%]		Statistical significance (p)
				**PCOS**	**Control**	**PCOS**	**Control**	
Açmaz et al. (2013), Turkey	BDI	BAI	No antidepressants, glucocorticoids or mood stabilizers	Hirsutism-acnea group: 24.46 ± 9.76<br>Infertility group: 30.59 ± 11.31<br>Obesity group: 19.10 ± 8.52<br>**Prevalence*: 90.70%**	12.28 ± 6.35<br>**Prevalence*: 53.19%**	Hirsutism-acnea group: 20.00 (IQR: 14.0–26.0)<br>Infertility group: 13.50 (IQR: 10.0–21.0)<br>Obesity group: 24.00 (IQR: 21.0–34.0)<br>Prevalence: not stated	12.00 (IQR: 9.0–16.0)<br>Prevalence: not stated	<0.001
		LSAS				Hirsutism-acnea group: 55.26 ± 12.30<br>Infertility group: 43.18 ± 11.17<br>Obesity group: 64.38 ± 13.24<br>Prevalence: not stated	37.98 ± 8.81<br>Prevalence: not stated	
Adali et al. (2008), Turkey	BDI	–	No oral contraceptives, glucocorticoids, antiandrogens, insulin sensitizers or psychiatric medications for at least 6 months	11.69 ± 9.49<br>**Prevalence*: 33.30%**	5.80 ± 4.58<br>**Prevalence*: 11.90%**	–	–	<0.050
Altinkaya et al. (2014), Turkey	BDI	BAI	No meds for at least 3 months	**Prevalence*: 38.00%**	**Prevalence*: 10.00%**	Not stated	Not stated	Not stated

Alur-Gupta et al. (2021), USA	HADS-D	HADS-A	None	White: 4.80 ± 3.60<br>**Prevalence*: 24.40%**<br>Black: 5.10 ± 4.00<br>**Prevalence: 29.00%**	White: 3.60 ± 3.30<br>Black: 4.10 ± 3.50<br>Prevalence: not stated	White: 10.30 ± 4.10<br>**Prevalence*: 75.90%**<br>Black: 8.70 ± 4.60<br>**Prevalence: 61.30%**	White: 8.10 ± 3.80<br>Black: 7.50 ± 4.80<br>Prevalence: not stated	Depressive symptoms: >0.050<br>Anxiety: <0.050
Arshad et al. (2012), Iran	BDI	–	None	**Prevalence*: 32.39%**	**Prevalence*: 14.18%**	–	–	Not stated
Asik et al. (2015), Turkey	HADS-D	HADS-A	None	6.10 ± 3.75<br>**Prevalence*: 42.25%**	3.54 ± 2.79<br>**Prevalence*: 14.00%**	8.59 ± 4.52<br>**Prevalence*: 35.21%**	5.98 ± 3.05<br>Prevalence: not stated	Depressive symptoms: <0.001<br>Anxiety: <0.010
Basirat et al. (2019), Iran	BDI-II	–	None	18.06 ± 12.03<br>Mild: 29.40%<br>Moderate: 27.70%<br>Severe: 16.80%<br>**Prevalence*: 73.90%**	15.65±11.76<br>Mild: 22.90%<br>Moderate: 28.00%<br>Severe: 10.10%<br>**Prevalence*: 61.00%**	–	–	>0.050
Batool et al. (2016), Pakistan	HADS-D	HADS-A	None	**Prevalence*: 29.93%**	**Prevalence*: 8.76%**	**Prevalence*: 15.33%**	**Prevalence*: 8.03%**	Depressive symptoms: <0.0001<br>Anxiety: <0.050
Battaglia et al. (2008), Italy	BDI	–	No psychoactive or hormone replacement medications for at least 6 months	Mild: 16.00%<br>Moderate: 4.00%<br>**Prevalence*: 20.00%**	Mild: 11.11%<br>Moderate: 5.56%<br>**Prevalence*: 16.67%**	–	–	>0.050
Bazarganipour et al. (2013), Iran	HADS-D	HADS-A	No medications for at least 3 months	**Prevalence*: 20.00%**	–	**Prevalence*: 47.00%**	–	Not stated

Benson et al. (2008), Germany	BDI	–	No psychiatric or hormonal replacement medications	9.70 ± 1.40<br>Mild: 40.63%<br>Moderate: 15.63%<br>**Prevalence*: 56.25%**	4.90 ± 0.9<br>Mild: 9.38%<br>Moderate: 0.00%<br>**Prevalence*: 9.38%**	–	–	<0.010
Bhattacharya et al. (2010), India	PHQ-9	–	No psychiatric or hormone replacement medications for at least 3 months	**Prevalence*: 64.10%**	**Prevalence*: 23.81%**	–	–	<0.0001
Cinar et al. (2011), Turkey	HADS-D<br>BDI	HADS-A<br>STAI	No medications for at least 3 months	**Prevalence*: 17.30%**<br>**Prevalence*: 28.32%**	Prevalence: not stated<br>**Prevalence*: 4.71%**	**Prevalence*: 42.30%**<br>Prevalence: not stated	Prevalence: not stated<br>Prevalence: not stated	<0.010
Cipkala-Gaffin et al. (2012), USA	BDI I	STAI	None	7.80 ± 7.00<br>Mild: 24.22%<br>Moderate: 4.97%<br>Severe: 1.86%<br>**Prevalence*: 31.06%**	5.70 ± 5.00<br>Mild: 14.91%<br>Moderate: 2.48%<br>Severe: 0.00%<br>**Prevalence*: 17.39%**	9.50 ± 5.50<br>Prevalence: not stated	8.70 ± 5.20<br>Prevalence: not stated	Depressive symptoms: <0.040<br>Anxiety: <0.010
Cirik et al. (2016), Turkey	HADS-D	HADS-A	No medications for at least 3 months	7.00 ± 4.00<br>Prevalence NIH-phenotype: 46.30%<br>Prevalence non-NIH-phenotype: 46.80%<br>**Prevalence*: 46.53%**	6.00 ± 2.00<br>**Prevalence*: 20.41%**	10.00 ± 5.00<br>Prevalence NIH-phenotype: 31.50%<br>Prevalence non-NIH-phenotype: 36.20%<br>**Prevalence*: 33.66%**	8.00 ± 3.00<br>**Prevalence*: 12.24%**	<0.050
Davari-Tanha et al. (2014), Iran	Mini-NPI by psychiatrist	–	No oral contraceptives, sodium	**Prevalence*: 80.00%**	**Prevalence*: 87.27%**	–	–	<0.050

			valproate or phenytoin medications					
Dybciak et al. (2022), Poland	HADS-D	HADS-A	None	Mild: 23.50%<br>Moderate: 13.90%<br>Severe: 4.30%<br>**Prevalence*: 41.70%**	Mild: 10.10%<br>Moderate: 6.00%<br>Severe: 0.00%<br>**Prevalence*: 16.10%**	Mild: 28.30%<br>Moderate: 26.00%<br>Severe: 20.00%<br>**Prevalence*: 74.30%**	Mild: 13.60%<br>Moderate: 21.10%<br>Severe: 4.50%<br>**Prevalence*: 39.20%**	<0.001
Ercan et al. (2013), Turkey	BDI	–	No oral contraceptives, estrogens, anti-androgens, sedatives, antidepressants, antidiabetic medications or beta blockers for at least 3 months	12.30 ± 4.10<br>**Prevalence*: 59.38%**	8.70 ± 2.70<br>**Prevalence*: 18.75%**	–	–	<0.001
Farzipour et al. (2024), Iran	BDI	BAI	No tricyclic antidepressants or anti-anxiety medications	**Prevalence*: 38.38%**	**Prevalence*: 15.79%**	**Prevalence: 4.04%**	**Prevalence: 6.32%**	Depressive symptoms: 0.003<br>Anxiety: >0.350
Glowinska et al. (2019), Poland	BDI	STAI	None	11.17 ± 7.65<br>Mild: 39.00%<br>Moderate: 9.76%<br>Severe: 2.44%<br>**Prevalence*: 51.20%**	–	42.88 ± 10.35<br>Prevalence: not stated	–	Not stated
Hebbar et al. (2023), UK, India	HADS-D	HADS-A	None	UK: 5.00 (IQR: 2.8–9.3)<br>India: 8.00 (IQR: 4.0–10.0)<br>Prevalence UK: 13.90%	–	UK: 11.00 (IQR: 7.8–12.3)<br>India: 12.00 (IQR: 8.0–14.0)<br>Prevalence UK: 50.00%	–	>0.050

				Prevalence India: 17.70% **Prevalence*: 16.50%**		Prevalence India: 59.50% **Prevalence*: 56.50%**		
Himelein et al. (2006), USA	BDI-SH	–	None	7.85 ± 7.00 **Prevalence*: 27.50%**	3.61 ± 4.08 **Prevalence*: 5.00%**	–	–	<0.001
Hollinrake et al. (2007), USA	PRIME-MD PHQ<br><br>BDI	–	None	**Prevalence*: 21.00%**<br><br>11.90 ± 11.10 **Prevalence*: 34.95%**	**Prevalence*: 3.00%**<br><br>4.50 ± 5.90 **Prevalence*: 10.68%%**	–	–	<0.010
Karsten et al. (2021), Netherlands	HADS-D	HADS-A	None	8.20 ± 3.70 **Prevalence*: 47.95%**	7.40 ± 3.20 **Prevalence*: 37.00%**	8.30 ± 3.90 **Prevalence*: 47.95%**	8.10 ± 3.40 **Prevalence*: 47.00%**	>0.050
Keeratibharat et al. (2024), Thailand	HADS-D	HADS-A	–	4.00 (IQR: 2.0–6.0) **Prevalence*: 3.85%**	–	6.67 ± 3.14 **Prevalence*: 11.92%**	–	Not stated
Kerchner et al. (2009), USA	PRIME-MD PHQ<br><br>BDI-II	PRIME-MD PHQ<br><br>BAI	None	**Prevalence*: 8.33%**<br><br>11.80 ± 9.30 **Prevalence*: 40.00%**	–	**Prevalence*: 15.00%**<br><br>8.40 ± 8.10 **Prevalence*: 23.30%**	–	<0.010
Kogure et al. (2019), Brazil	HADS-D	HADS-A	No hormone replacement or anti-androgenic medications for at least 3 months	6.70 ± 3.80 **Prevalence*: 32.10%**	–	8.60 ± 3.90 **Prevalence*: 57.10%**	–	<0.050
Mannson et al. (2008), Sweden	Mini-NPI by psychiatrist	Mini-NPI by psychiatrist	None	**Prevalence*: 67.35%**	**Prevalence*: 34.69%**	**Prevalence: 12.24%**	**Prevalence: 2.04%**	Depressive symptoms: <0.005 Anxiety:

								>0.050
Moran et al. (2012), Australia	HADS-D	HADS-A	No hormone replacement or insulin-sensitizing medication for at least 3 months	Mild: 27.00%<br>Moderate: 6.00%<br>Severe: 0.00%<br>**Prevalence*: 33.00%**	Mild: 8.00%<br>Moderate: 4.00%<br>Severe: 0.00%<br>**Prevalence*: 12.00%**	Mild: 19.00%<br>Moderate: 35.00%<br>Severe: 8.00%<br>**Prevalence*: 62.00%**	Mild: 42.00%<br>Moderate: 4.00%<br>Severe: 0.00%<br>**Prevalence*: 46.00%**	<0.050
Radhakrishnan et al. (2018), India	HADS-D	HADS-A	None	**Prevalence*: 16.00%**	–	**Prevalence*: 36.00%**	–	Not stated
Rassi et al. (2010), Brazil	Mini-NPI	Mini-NPI	None	**Prevalence*: 26.39%**	–	**Prevalence*: 9.72%**	–	Not stated
Soyupek et al. (2010), Turkey	BDI	BAI	None	8.92 ± 7.73<br>**Prevalence*: 30.00%**	5.25 ± 4.19<br>**Prevalence*: 10.30%**	4.22 ± 2.94<br>**Prevalence*: 5.00%**	1.89 ± 2.10<br>**Prevalence*: 0.00%**	Depressive symptoms: <0.050<br>Anxiety: <0.005
Tan et al. (2017), China	BDI	STAI	No psychiatric medications	12.10 ± 7.30<br>**Prevalence*: 27.50%**	7.80 ± 5.30<br>**Prevalence*: 3.00%**	State: 42.70 ± 11.70<br>Trait: 43.40 ± 9.80<br>**Prevalence: 13.30%**	State: 34.20 ± 10.10<br>Trait: 36.10 ± 9.40<br>**Prevalence: 2.00%**	Depressive symptoms: <0.001<br>Anxiety: >0.050
Tariq et al. (2021), Pakistan	HADS-D	HADS-A	No psychiatric medications	**Prevalence*: 60.00%**	**Prevalence*: 8.89%**	**Prevalence*: 85.93%**	**Prevalence*: 37.04%**	Depressive symptoms: <0.001<br>Anxiety: <0.005
Zueff et al. (2015), Brasil	HADS-D	HADS-A	No hormone replacement, anti-convulsant, or anti-androgenic medications for at least 12 months	**Prevalence*: 29.50%**	**Prevalence*: 18.70%**	**Prevalence*: 51.20%**	**Prevalence*: 52.30%**	>0.050

Note. *results included in the final analysis; BAI – Beck Anxiety Inventory; BDI – Beck Depression Inventory (BDI); HADS – Hospital Anxiety and Depression Scale; IQR – the interquartile range; LSAS – Liebowitz' Social Anxiety Scale; Mini-NPI – Mini-International Neuropsychiatric Interview; PHQ-9 – Primary Care Evaluation of Mental Disorders Patient Health Questionnaire 9; PRIME-MD PHQ – Primary Care Evaluation of Mental Disorders Patient Health Questionnaire; STAI – State-Trait Anxiety Inventory; UK – United Kingdom; USA – United States of America.
